# The role of digital device use on the risk of migraine: a univariable and multivariable Mendelian randomization study

**DOI:** 10.3389/fneur.2024.1462414

**Published:** 2024-10-30

**Authors:** Zongqing He, Fan Qiu, Jing Yang, Min Zhao

**Affiliations:** ^1^Center of Encephalopathy, The First Affiliated Hospital of Henan University of Chinese Medicine, Zhengzhou, China; ^2^Department of First Clinical Medical College, Henan University of Chinese Medicine, Zhengzhou, China; ^3^Department of Zhongjing College, Henan University of Chinese Medicine, Zhengzhou, China

**Keywords:** digital device, migraine, univariable Mendelian randomization study, multivariable Mendelian randomization study, casual effect

## Abstract

**Background:**

The pervasive integration of digital devices into daily life has raised concerns about their potential health impacts. This study aimed to explore the causal relationships between digital device use and the risk of migraine using Mendelian randomization (MR).

**Methods:**

Genetic data on digital device use and migraines were sourced from large-scale genome-wide association studies conducted by the UK Biobank, the FinnGen study, and the International Headache Genetics Consortium. Univariable MR (UVMR), meta-analysis, and multivariable MR (MVMR) approaches were conducted to explore and verify the causal effects of digital device use (including mobile phone use, computer use, playing computer games, and watching television) on migraine risk. Sensitivity analyses were conducted using Cochran’s Q, MR-Egger intercept test, MR pleiotropy residual sum and outlier, MR Radial, MR Steiger, and leave-one-out methods.

**Results:**

UVMR analyses revealed that genetically predicted mobile phone use was significantly associated with an increased risk of overall migraine (odds ratio [OR] = 2.39, *p* = 9.78e-5) and migraine without aura (MO) (OR = 2.25, *p* = 0.024). Additionally, there were significant positive associations between genetically predicted television watching and the risk of overall migraine (OR = 1.63, *p* = 2.12e-5) and MO (OR = 2.10, *p* = 4.98e-5). These results were further supported by the meta-analysis and MVMR analysis. Sensitivity analysis indicated no heterogeneity or pleiotropy.

**Conclusion:**

This comprehensive MR study provides preliminary evidence for the causal impact of mobile phone use and television watching on the risk of migraines. Further studies are needed to explore these associations across different populations.

## 1 Introduction

Migraine is a prevalent and debilitating neurological disorder characterized by recurring headaches, frequently accompanied by nausea, vomiting, and light and sound sensitivity (1). Affecting over 100 million people worldwide, primarily those under 50, it is the second leading cause of years lived with disability globally across all age groups (2, 3). Given its severe physical and mental impact on patients, preventing migraine attacks is crucial. Previous studies have identified several risk factors, including sleep patterns, dietary habits, physical activity, and medication use, which contribute to migraine (4). Thus, identifying additional triggers and developing strategies to mitigate them are essential for migraine prevention.

With continuous technological advancements, electronic devices have gradually integrated into our lives, becoming an indispensable part of modern life. Existing studies have found that artificial intelligence equipped with digital devices plays an important role in the diagnosis, prevention, and management of migraines (5). Although these devices offer significant convenience in medicine, work, and entertainment, their use also results in prolonged screen time and sedentary behavior, posing potential health risks (6). Digital addiction, which is closely related to genetic predisposition (7), significantly affects brain function and structure (8). Prolonged exposure to blue light and electromagnetic radiation may cause neurological dysfunctions such as headaches, sleep disorders, negative emotions, memory decline, and attention deficits (6). Observational studies have found that frequent use of electronic devices is associated with an increased risk of migraine, particularly among students (9, 10). However, traditional observational studies are prone to interference from confounding factors, which limit the reliability of establishing causal relationships, thus making it difficult to establish a clear causal relationship between digital device use and migraine risk.

Mendelian randomization (MR) is an epidemiological method used to assess causal inference by utilizing genetic variations strongly associated with the exposure of interest as instrumental variables (IVs) (11). Currently, MR is being increasingly applied in clinical research to effectively predict drug efficacy, optimize experimental designs, and expand feasibility trials (12–14). Since genetic variations are present at birth and remain stable throughout life, MR analysis results are less likely to be influenced by reverse causation and confounders. Therefore, we utilized a comprehensive MR analysis to explore the causal effects of electronic device use on migraine (15, 16).

## 2 Materials and methods

### 2.1 Study design

Three core assumptions ensure the validity of MR results (17). First, the relevance assumption requires genetic variants to be strongly associated with the exposure of interest. Second, the independence assumption ensures these genetic variants are free from confounders that could affect the exposure–outcome relationship. Third, the exclusion restriction assumption requires the genetic variants to influence the outcome only through exposure, not via any other pathways.

**Figure 1:**
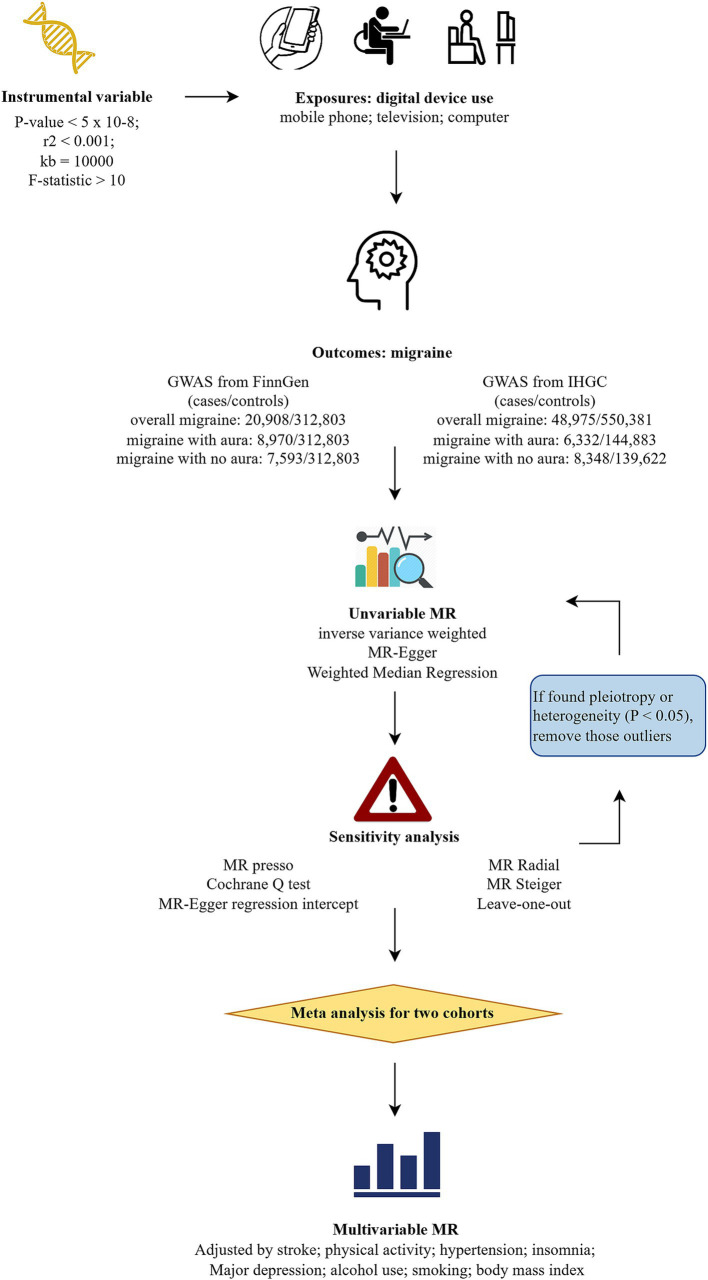
Flowchart of study design. MR, Mendelian randomization; GWAS, genome-wide association studies; IHGC, international headache genetics consortium; MR-PRESSO, Mendelian randomization pleiotropy residual sum and outlier.

Figure 1 illustrates the study design. Initially, we conducted univariable MR (UVMR) to assess the causal relationship between digital device use and the risk of overall migraine and its subtypes, using data from two separate genome-wide association studies (GWAS). Subsequently, we performed a meta-analysis to combine the results, followed by multivariable MR (MVMR) to account for potential confounders, involving stroke, hypertension, physical activity levels, smoking, alcohol consumption, body mass index, insomnia, and major depression (18, 19).

All GWAS data were sourced from publicly accessible repositories, ensuring transparency and reproducibility. Ethical approval was obtained for the original GWAS data used in this study, adhering to ethical standards and guidelines governing such research. This study was reported in accordance with STrengthening the Reporting of OBservational studies in Epidemiology-Mendelian Randomization (STROBE-MR) guidelines (Supplementary Table 1.1) (20).

### 2.2 Data source

#### 2.2.1 Genome-wide association studies data for digital device use

The GWAS data on four types of digital device use, including mobile phone use, television watching, computer use, and playing computer games, were obtained from the UK Biobank (Supplementary Table 2.1). The UK Biobank is a large-scale biomedical database and research resource containing comprehensive genetic and health data from half a million UK participants and is widely used in various health-related research (21). Digital device use was based on self-reported data. Mobile phone use was defined as the frequency of making or receiving calls by mobile phone per week over the past 3 months (*n* = 386,626 participants). Television watching was measured by daily viewing time (*n* = 437,887), computer use was assessed through daily computer usage time (*n* = 360,895), and computer gaming habits were evaluated based on gaming practices (*n* = 462,433).

#### 2.2.2 Genome-wide association studies data for migraine

The GWAS data on migraine were sourced from two large datasets. FinnGen was the primary discovery cohort, a public–private partnership project in Finland that combines genetic data with digital health records from national health registries (22). Migraine phenotypes were classified based on the International Classification of Diseases (ICD 10) code: R10, comprising 20,908 cases of overall migraine, 8,970 cases with aura (MA), and 7,593 cases without aura (MO). The replication cohort, from the International Headache Genetics Consortium (IHGC), comprises 48,975 European cases of overall migraine excluding the 23andMe cohort owing to permission restrictions (23). MA and MO cases included 6,332 and 8,348 European cases, respectively (24). Migraine cases were characterized by clinical phenotyping or self-reported information in IHGC. Detailed information is provided in Supplementary Table 2.1. We did not interpolate missing data for the GWAS in this study.

### 2.3 Genetic instrument selection

To select robust IVs, only single nucleotide polymorphisms (SNPs) with a *p*-value <5e-8 and minor allele frequencies >0.01 were selected. SNP independence was ensured using the 1,000 Genomes Project European reference panel, applying the linkage disequilibrium (LD) criteria of r^2^ < 0.001 within a 10 Mb window (25). F-statistics were calculated to assess the strength of each SNP. F-statistic >10 was considered a strong instrument (26). The formulas for calculating the F-statistic and R^2^ were as follows, where, N is = sample size, and k is = number of IVs.


$$F=\frac{R^{2}\left(N-k-1\right)}{k\left(1-R^{2}\right)}$$

$$R^{2}=\frac{{\mathrm{BETA}}^{2}}{\left({\mathrm{BETA}}^{2}+{SE}^{2}\right)N}$$

### 2.4 Univariable Mendelian randomization and sensitivity analysis

The effect alleles were aligned between the GWAS datasets for digital device use and migraine. The UVMR approach was conducted to examine the potential causality between digital device use and migraine risk using three methods. The inverse-variance weighted (IVW) method was the primary analysis, offering the most precise estimates when assuming no horizontal pleiotropy among genetic instruments (27). MR-Egger regression allowed for the detection and correction of pleiotropy, accounting for the average pleiotropic effect of the instruments (28). The weighted median method provided a robust causal estimate even when up to 50% of the genetic instruments were invalid, calculating the median of ratio estimates, weighted by their variances (29). Causality was considered stable if the three methods had consistent results, with scatter plots used to illustrate these results.

Detecting pleiotropy and heterogeneity is crucial in studies to ensure that IVs satisfy the core assumptions of valid causal inference. Therefore, several sensitivity analyses were used to validate the robustness of the MR analyses. The Mendelian randomization pleiotropy residual sum and outlier (MR-PRESSO) method was used to detect and correct pleiotropy by removing outliers, while MR-Egger regression estimated the intercept to detect pleiotropic bias. A non-zero intercept indicated the presence of directional pleiotropy (30, 31). Cochran’s Q test was calculated using MR-Egger and IVW methods to assess heterogeneity among the genetic instruments (32). MR Radial was conducted to further detect and correct outliers. MR and sensitivity analyses were repeated after removing these outliers (33). Furthermore, the MR Steiger test was employed to estimate the potential reverse causality between digital devices and migraine (34). Leave-one-out (LOO) analysis was performed to detect any pleiotropy driven by a single SNP.

### 2.5 Meta-analysis of the estimates

A meta-analysis was conducted to combine the causal estimates derived from the IVW analyses of both the discovery and replication datasets, subsequently validating the causal association between digital device use and migraine. When the I^2^ value exceeded 50%, a random-effects model was utilized to combine the results. Otherwise, fixed-effects models were applied (35).

### 2.6 Multivariate Mendelian randomization

MVMR was performed utilizing the IVW method to clarify the independent effects of digital device usage on migraine accounting for potential confounders including stroke, hypertension, physical activity levels, smoking, alcohol consumption, body mass index, insomnia, and depression (36).

### 2.7 Statistic analysis

The associations between genetically predicted digital device use and the risk of migraine were presented as odds ratios (ORs) with 95% confidence intervals (CIs). All analyses were conducted using R software (version 4.3.1). MR analyses were performed using the TwoSampleMR package, and meta-analyses were conducted using the meta package.

Considering the likelihood of false positives, the Bonferroni method was conducted for multiple testing corrections. A *p*-value <0.0042 (0.05/3/4) was considered statistically significant evidence of a causal relationship, while *p*-values <0.05 but above the Bonferroni-corrected threshold indicated a potential causal association. In this study, we used the generative AI technology ChatGPT (version: GPT-4, model: GPT-4 (2023), source: https://openai.com/) provided by OpenAI to assist in translation and editing of the manuscript.

## 3 Results

For digital device use, the F-statistics for IVs were all greater than 10, ranging from 29.7 to 151.8, indicating no weak instrument bias (Supplementary Tables 3.1–3.4). The average *F*-values for the SNPs related to the four types of devices were as follows: mobile phone use (38.0), computer use (38.1), playing computer games (39.5), and television watching (41.5).

### 3.1 Discovery results of univariate Mendelian randomization

The IVW estimates revealed that genetically predicted mobile phone use was associated with an increased risk of overall migraine (OR = 2.39, 95% CI 1.54–3.70; *p* = 9.78e-5) and MO (OR = 2.25, 95% CI 1.11–4.53; *p* = 0.024). Similarly, television watching was positively associated with an increased risk of overall migraine (OR = 1.63, 95% CI 1.30–2.04; *p* = 2.12e-5) and MO (OR = 2.10, 95% CI 1.47–3.01; *p* = 4.98e-5), but neither was significantly associated with MA.

Negative associations were observed between computer use (OR = 0.67, 95% CI 0.46, 0.97; *p* = 0.035) and playing computer games (OR = 0.41, 95% CI 0.18, 0.91; *p* = 0.028) with MO, though neither was significantly associated with overall migraine or MA. After Bonferroni correction, mobile phone use was significant with an increased risk of overall migraine, while television watching was significant for both overall migraine and MO. All results of UVMR are presented in Supplementary Tables 2.2–2.4, and scatter plots are shown in Supplementary Figures S4–S6.

No significant pleiotropy or heterogeneity was detected after excluding outlier SNPs, indicating a robust causal inference and alignment with core MR assumptions. The detailed results of the pleiotropy and heterogeneity tests are presented in Supplementary Tables 2.5, 2.6, while information on outlier SNPs is presented in Supplementary Tables 2.10, 2.11 of the same article. The LOO analysis suggested that our findings were not driven by any single SNP, indicating the robustness of the causality between the use of digital devices and migraines. Additionally, no individual SNP significantly altered the overall conclusion, further supporting the reliability of our MR results (Supplementary Figures S1–S3).

### 3.2 Combined results for migraine from a meta-analysis

**Figure 2:**
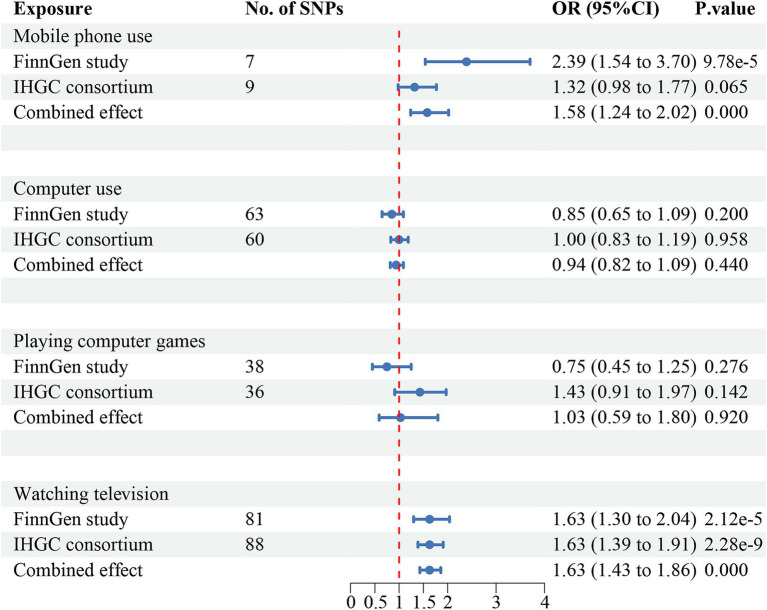
Causal association between digital device use and overall migraine. Estimated ORs for the effect of digital device use on migraine, obtained from an IVW analysis, per outcome database separately and combined over the two databases using meta-analyses. CI, confidence interval; SNPs, single-nucleotide polymorphisms.

**Figure 3:**
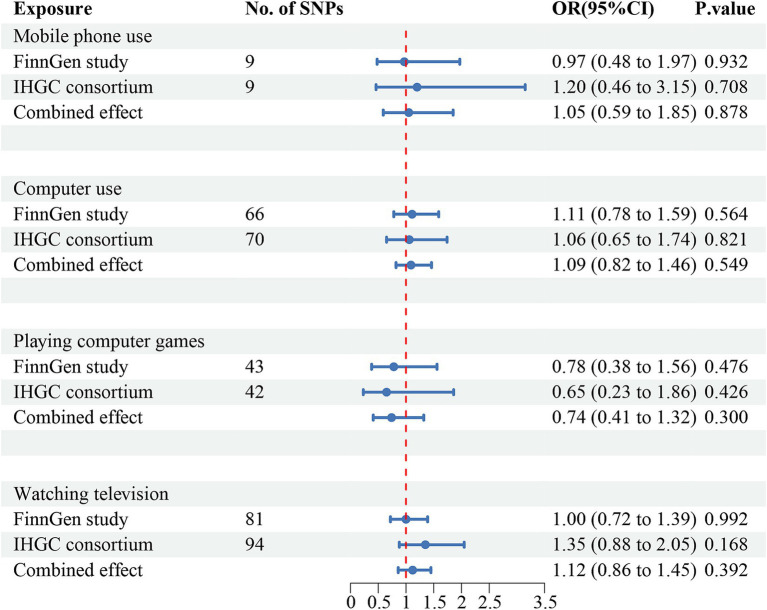
Causal association between digital device use and migraine with aura. Estimated ORs for the effect of digital device use on migraine, obtained from an IVW analysis, per outcome database separately and combined over the two databases using meta-analyses. CI, confidence interval; SNPs, single-nucleotide polymorphisms.

**Figure 4:**
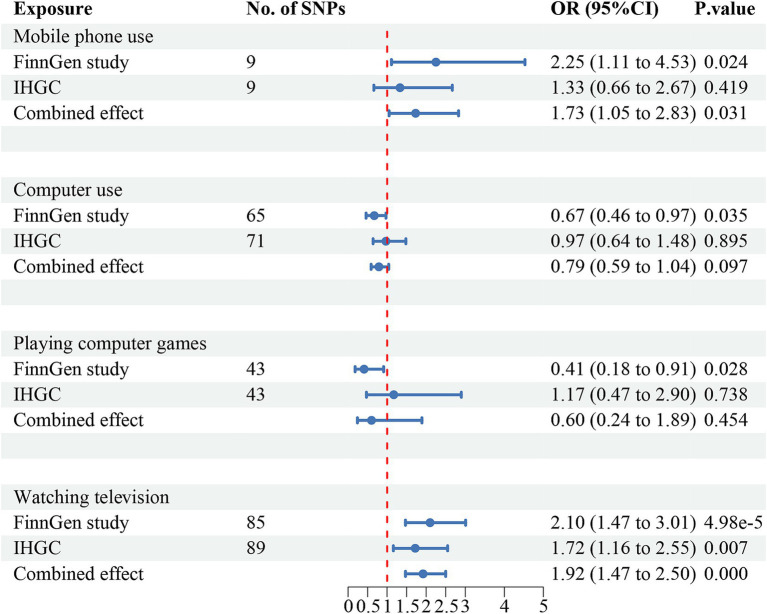
Causal association between digital device use and migraine with no aura. Estimated ORs for the effect of digital device use on migraine, obtained from an IVW analysis, per outcome database separately and combined over the two databases using meta-analyses. CI, confidence interval; SNPs, single-nucleotide polymorphisms.

The meta-analysis of the causal effects of digital device use on overall migraine, MA, and MO are shown in Figures 2–4, respectively. The IVW estimates from two separate datasets verified a significant causal association between mobile phone use and overall migraine (OR = 1.58, 95% CI 1.24, 2.02; *p* = 0.000) and suggestive evidence for MO (OR = 1.73, 95% CI 1.05, 2.83; *p* = 0.031) Television watching was significantly associated with overall migraine (OR = 1.63, 95% CI 1.43, 1.86; *p* = 0.000) and MO (OR = 1.92, 95% CI 1.47, 2.50, *p* = 0.000). No causal relationships were found between computer use, playing computer games, and any migraine subtypes.

### 3.3 Multivariate Mendelian randomization

MVMR analysis, adjusted for relevant confounders, confirmed that watching television independently increased the risk of migraine (OR = 2.01, 95% CI 1.32, 3.07; *p* = 0.001) and MO (OR = 3.56, 95% CI 1.90, 6.66; *p* = 6.99e-5). Similarly, mobile phone use was independently associated with an increased risk of migraine (OR = 1.40, 95% CI 1.03, 1.90; *p* = 0.032) and MO (OR = 1.88, 95% CI 1.20, 2.96; *p* = 0.006). These results are consistent with those from the UVMR analysis and the meta-analysis (Supplementary Tables 2.7–2.9).

## 4 Discussion

This study employed comprehensive MR analysis to investigate the causal relationships between the use of various digital devices and the risk of migraine and its subtypes. The findings suggested potential adverse effects of frequent mobile phone use and prolonged television watching on migraine risk, particularly in individuals with migraine without aura. However, no robust evidence was observed for causality between computer use or playing video games and migraine or its subtypes.

### 4.1 Comparison with previous studies

Previous observational studies have indicated the detrimental effects of excessive electronic device use on migraine, especially among younger populations (37–40). For instance, a cross-sectional study conducted in Saudi Arabia involving 504 medical students found that using various electronic devices for ≥4 h daily was associated with a higher risk of headaches compared to those with <4 h daily. Notably, over 70% of students reported that reducing or stopping the use of electronic device use helped alleviate their headache symptoms (10). Our study builds on this evidence by providing potential causal inferences for mobile phone use and television watching on migraine, however, there is no consistent causal relationship between computer use and playing video games.

For mobile phone use, in line with our MR findings, a meta-analysis that combined the results of 30 cohorts involving multiple ethnicities and populations aged 9–63 years found a positive correlation between weekly mobile phone usage and the risk of migraines, suggesting that mobile phone radiation may be a risk factor for migraine (41). Additionally, a large Danish nationwide prospective cohort study investigated whether mobile phone usage was correlated with neurological disorders and found that increased mobile phone usage raised the consultation rates for migraine and dizziness (42). Similarly, a cross-sectional study by Butt et al. (43), involving approximately 400 patients experiencing migraine aged 18–65 years without other neurological diseases found that prolonged smartphone use was linked to increased migraine duration and frequency. Similarly, Brindova et al. (44) found that watching television for more than 3 h daily was correlated with an elevated incidence of headaches in adolescents. Consistent with another MR study (14), our results indicate an adverse effect of genetically predicted television watching on migraine risk, particularly for MO.

Regarding computer use and playing video games, observational evidence of computer use on elevated migraine risk was provided in a workplace study conducted in the Philippines (45). Additionally, a cross-sectional study among Peruvian medical students indicated that playing computer games could increase the probability of migraine (46). Langdon et al. (47) reviewed electronic device types linked to headache triggers and concluded that extended computer use and video gaming are common migraine triggers in children. However, our MR analyses do not support these claims, as we found no significant causal relationship between computer use or video gaming and migraine. These inconsistencies may be attributed to differences in the ages of the study populations, the motivations for device usage, and environmental factors across different studies. For example, computer use in the workplace often involves more work-related stress than computer use for entertainment activities (46). PC gaming or computer use may impose more burden on migraines in adolescents than adults (48).

### 4.2 Potential mechanism

The connection between electronic device use and migraines can be explained by several mechanisms, prolonged exposure to blue light, and electromagnetic radiation. A clinical trial found that migraine patients exhibited a significant and sustained decrease in pain perception thresholds following light stimulation compared to healthy individuals (49). This phenomenon may be attributed to blue light’s stimulation of intrinsically photosensitive retinal ganglion cells, which subsequently affects the conduction of the trigeminal nociceptive pathway. These findings suggest that visual stimuli could trigger migraine (50). In addition, blue light exposure can disrupt sleep and maintain brain alertness, especially excessive use of electronic devices before bedtime, which imposes a burden on migraine (51–53).

The nervous system is highly sensitive to electromagnetic radiation. Prolonged exposure can lead to neurotransmitter metabolism disorders and oxidative stress in central nervous system cells (8), both of which are closely linked to migraine pathogenesis. An epidemiological survey found that among 293 French individuals with electromagnetic hypersensitivity (EHS), the prevalence of migraine was approximately 65% (54). Another study conducted in Thailand found that electromagnetic radiation emitted by smartphones may be one of the triggers of migraines among adolescents, considering that this radiation could affect the opioid receptor system and reduce the pain threshold (55). Studies have found that long-term exposure to electromagnetic radiation can cause metabolic disorders of amino acids such as glutamate and GABA in brain tissue, the balance between excitation and inhibition within the central nervous system. This imbalance may lead to the abnormal activation of the pain perception system, triggering migraine (56, 57). Moreover, the abnormal expression of serotonin has also been reported to be affected by microwave radiation (58, 59), which may cause local vasodilation, thereby triggering migraine.

Additionally, an imbalance between oxidants and antioxidants in the brain leads to oxidative stress during exposure to microwave radiation (60), which induces inflammation and triggers migraines (61). A case report showed that a patient with EHS experienced severe migraine symptoms after exposure to digital devices, including cell phones and television, and further examination showed elevated levels of circulating antibodies against oxidized low-density lipoprotein (LDLox), a marker of oxidative stress (62). The mechanism underlying the impact of electronic devices on migraines is rather complex and multifaceted, requiring further research.

Several factors related to electronic device usage may influence its causal relationship with device use. Prolonged screen time has been shown to affect mental health, leading to insomnia and depression (63). Additionally, excessive electronic device use is often associated with poor lifestyle habits such as a lack of physical activity and prolonged sedentary behavior, contributing to obesity, hypertension, and cardiovascular diseases. These behaviors are often linked to unhealthy dietary habits, including smoking and excessive alcohol consumption (64). Both lifestyle and mental health factors commonly associated with digital device use are recognized as risk factors for migraines (65). Our MVMR analysis underlined the independent effects of smartphone use and watching television on migraines after accounting for these confounders.

### 4.3 Strengths and limitations

Our study has several strengths. First, we employed UVMR analysis using SNPs as IVs to assess the causal effects of electronic device usage on migraine risk. This method minimizes confounding factors since alleles are randomly allocated to offspring during fertilization. Additionally, we increased the statistical power and reliability of our findings by combining data from two large GWAS databases. Through MVMR, we further explored the independent causal relationship between digital device use and migraines. Additionally, multiple sensitivity analyses were conducted to clarify the reliability of the findings, which revealed no significant pleiotropy or heterogeneity.

Despite these strengths, our MR study has some limitations. First, while the MR analysis method aims to reduce confounding factors, some unmeasured confounders and weak IVs may still influence the outcomes (12). However, we used strict IV selection criteria, and the MVMR helped adjust for some pleiotropic factors related to migraine, minimizing these risks of bias. Second, due to the inherent limitations associated with GWAS data, exposure and outcome phenotypes rely on self-reported data, which may lead to recall bias. Additionally, the non-linear associations between digital device usage and migraine could not be assessed in this study. Third, the GWAS data used in this study were from European populations, lacking diversity in sample composition. Without subgroup analyses based on age and sex, these results may not be relevant to other demographic groups. Furthermore, there was a lack of information on other digital device usage, including tablets and laptops, as well as the specific contexts and motivations for electronic device usage. Future research should refine the categories, motivations, and environments surrounding electronic device use to provide a clearer understanding of the factors contributing to migraine risk.

## 5 Conclusion

In conclusion, our study provides evidence of the possible causal relationship between frequent mobile phone use, television watching, and migraine risk, particularly migraine without aura. Future research is needed to validate these associations in non-European populations and across different age or sex groups to ensure broader generalizability.

## Data Availability

The original contributions presented in the study are included in the article/Supplementary material, further inquiries can be directed to the corresponding author.
