# Supplementary material for: The role of digital device use on the risk of migraine: a univariable and multivariable Mendelian randomization study
Source: Front Neurol. 2024 Oct 30;15:1462414. doi: 10.3389/fneur.2024.1462414 (PMC11557339; doi:10.3389/fneur.2024.1462414)
Supplement: Supplementary file 2 [file Table_2.docx]

**Table 2, Supplementary Tables 2.1 to 2.11**

[**Supplementary Table 2.1. Details of data sources of the GWAS included in the Mendelian randomization** 2](#_Toc180582372)

[**Supplementary Table 2.2. UVMR estimates for the associations between digital device use and overall migraine** 3](#_Toc180582373)

[**Supplementary Table 2.3. UVMR estimates for the associations between digital device use and migraine with aura** 5](#_Toc180582374)

[**Supplementary Table 2.4. UVMR estimates for the associations between digital device use and migraine with no aura** 7](#_Toc180582375)

[**Supplementary Table 2.5. Heterogeneity and Horizontal pleiotropy of MR analysis** 9](#_Toc180582376)

[**Supplementary Table 2.6. Heterogeneity and Horizontal pleiotropy of initial MR analysis** 11](#_Toc180582377)

[**Supplementary Table 2.7. Estimates from MVMR between digital devices use and migraine** 13](#_Toc180582378)

[**Supplementary Table 2.8. Estimates from MVMR between digital devices use and migraine with aura** 14](#_Toc180582379)

[**Supplementary Table 2.9. Estimates from MVMR between digital devices use and migraine with no aura** 15](#_Toc180582380)

[**Supplementary Table 2.10. Removed outliers of UVMR in discovery corhort** 16](#_Toc180582381)

[**Supplementary Table 2.11. Removed outliers of UVMR in validation corhort** 17](#_Toc180582382)

**Supplementary Table 2.1. Details of data sources of the GWAS included in the Mendelian randomization**

| Phenotype | Study type | Samplesize | Total No. of SNPs | Population | Consortium/PubMed ID | Web Source |
| --- | --- | --- | --- | --- | --- | --- |
| Weekly usage of mobile phone in last 3 months | GWAS | 386,626 | 9,851,867 | European | MRC-IEU | IEU: ukb-b-17999 |
| Time spent computer using | GWAS | 360,895 | 9,851,867 | European | MRC-IEU | IEU: ukb-b-4522 |
| Plays computer games | GWAS | 462,433 | 9,851,867 | European | MRC-IEU | IEU: ukb-b-4779 |
| Time spent watching television | GWAS | 437,887 | 9,851,867 | European | MRC-IEU | IEU: ukb-b-5192 |
| Overall Migraine | Meta-analysis | 599,356 | 11,380,305 | European | 35115687 | d.nyholt@qut.edu.au |
|  | GWAS | 333,711 | 20,168,276 | European | FinnGen study | www.finngen.fi/en |
| Migraine with aura | Meta-analysis | 151,215 | 8,809,897 | European | 27322543 | if.iknisleh@cad-mmif |
|  | GWAS | 321,773 | 20,167,894 | European | FinnGen study | www.finngen.fi/en |
| Migraine without aura | Meta-analysis | 147,970 | 6,701,886 | European | 27322543 | if.iknisleh@cad-mmif |
|  | GWAS | 320,396 | 20,167,888 | European | FinnGen study | www.finngen.fi/en |
| Stroke | Meta-analysis | 446,696 | 7,633,440 | European | 29531354 | IEU: ebi-a-GCST005838 |
| Moderate to vigorous physical activity levels | GWAS | 377,234 | 11,808,007 | European | 29899525 | IEU: ebi-a-GCST006097 |
| Hypertension | GWAS | 484,598 | 9,587,836 | European | 33959723 | IEU: ebi-a-GCST90038604 |
| Major depression | GWAS | 500,199 | -- | European | PGC | IEU: ieu-b-102 |
| Alcoholic drinks per week | GWAS | 335,394 | 11,887,865 | European | GSCAN | IEU: ieu-b-73 |
| Cigarettes smoked per day | GWAS | 249,752 | 12,003,613 | European | GSCAN | IEU: ieu-b-142 |
| Body mass index | GWAS | 461,460 | 9,851,867 | European | MRC-IEU | IEU: ukb-b-19953 |
| Sleeplessness / insomnia | GWAS | 462,341 | 9,851,867 | European | MRC-IEU | IEU: ukb-b-3957 |

GWAS, genome-wide association study.

**Supplementary Table 2.2. UVMR estimates for the associations between digital device use and overall migraine**

| Exposure | Outcome | Data source | Method | nSNPs | Beta | Se | P-value | OR | OR_lci95 | OR_uci95 |
| --- | --- | --- | --- | --- | --- | --- | --- | --- | --- | --- |
| Mobile phone use | Overall migraine | FinnGen | MR Egger | 7 | 0.3523 | 1.3533 | 0.8050 | 1.4223 | 0.1002 | 20.1824 |
|  |  |  | Weighted median | 7 | 0.9506 | 0.3054 | 0.0019 | 2.5872 | 1.4220 | 4.7074 |
|  |  |  | Inverse variance weighted | 7 | 0.8698 | 0.2233 | **9.78e-5** | 2.3863 | 1.5406 | 3.6963 |
|  |  | IHGC | MR Egger | 9 | 0.3716 | 0.9394 | 0.7042 | 1.4500 | 0.2300 | 9.1414 |
|  |  |  | Weighted median | 9 | 0.2425 | 0.1988 | 0.2225 | 1.2744 | 0.8632 | 1.8815 |
|  |  |  | Inverse variance weighted | 9 | 0.2765 | 0.1495 | 0.0645 | 1.3185 | 0.9835 | 1.7675 |
| Computer use | Overall migraine | FinnGen | MR Egger | 63 | 0.0554 | 0.7691 | 0.9429 | 1.0569 | 0.2341 | 4.7725 |
|  |  |  | Weighted median | 63 | -0.1507 | 0.1798 | 0.4022 | 0.8601 | 0.6046 | 1.2237 |
|  |  |  | Inverse variance weighted | 63 | -0.1678 | 0.1309 | 0.1998 | 0.8456 | 0.6543 | 1.0928 |
|  |  | IHGC | MR Egger | 60 | 0.4913 | 0.5209 | 0.3495 | 1.6344 | 0.5888 | 4.5366 |
|  |  |  | Weighted median | 60 | 0.0624 | 0.1351 | 0.6442 | 1.0644 | 0.8167 | 1.3872 |
|  |  |  | Inverse variance weighted | 60 | -0.0047 | 0.0904 | 0.9584 | 0.9953 | 0.8336 | 1.1883 |
| Plays computer games | Overall migraine | FinnGen | MR Egger | 38 | -0.4773 | 1.4421 | 0.7426 | 0.6205 | 0.0367 | 10.4781 |
|  |  |  | Weighted median | 38 | -0.1390 | 0.3709 | 0.7080 | 0.8703 | 0.4206 | 1.8005 |
|  |  |  | Inverse variance weighted | 38 | -0.2827 | 0.2593 | 0.2755 | 0.7537 | 0.4534 | 1.2529 |
|  |  | IHGC | MR Egger | 36 | -0.1796 | 0.9742 | 0.8549 | 0.8356 | 0.1238 | 5.6404 |
|  |  |  | Weighted median | 36 | 0.2793 | 0.2701 | 0.3011 | 1.3222 | 0.7787 | 2.2448 |
|  |  |  | Inverse variance weighted | 36 | 0.2900 | 0.1977 | 0.1425 | 1.3364 | 0.9071 | 1.9690 |
| Watching television | Overall migraine | FinnGen | MR Egger | 79 | 0.7760 | 0.5996 | 0.1995 | 2.1728 | 0.6709 | 7.0371 |
|  |  |  | Weighted median | 79 | 0.4530 | 0.1693 | **0.0075** | 1.5731 | 1.1288 | 2.1923 |
|  |  |  | Inverse variance weighted | 79 | 0.4873 | 0.1146 | **2.12e-5** | 1.6279 | 1.3004 | 2.0379 |
|  |  | IHGC | MR Egger | 88 | 0.4756 | 0.3582 | 0.1878 | 1.6090 | 0.7973 | 3.2470 |
|  |  |  | Weighted median | 88 | 0.5131 | 0.1181 | **1.40e-5** | 1.6704 | 1.3252 | 2.1056 |
|  |  |  | Inverse variance weighted | 88 | 0.4880 | 0.0817 | **2.28e-9** | 1.6290 | 1.3881 | 1.9118 |
| UVMR, univariable mendelian randomization; SNPs, single-nucleotide polymorphisms; Beta, beta coefficient; Se, standard error; OR, odds ratio; lci, lower confidence interval; uci, upper confidence interval. | | | | | | | | | | |

**Supplementary Table 2.3. UVMR estimates for the associations between digital device use and migraine with aura**

| Exposure | Outcome | Data source | Method | nSNPs | Beta | se | P-value | OR | OR_lci95 | OR_uci95 |
| --- | --- | --- | --- | --- | --- | --- | --- | --- | --- | --- |
| Mobile phone use | MA | FinnGen | MR Egger | 9 | -3.3060 | 1.9482 | 0.1335 | 0.0367 | 0.0008 | 1.6695 |
|  |  |  | Weighted median | 9 | 0.0798 | 0.4059 | 0.8442 | 1.0830 | 0.4888 | 2.3997 |
|  |  |  | Inverse variance weighted | 9 | -0.0308 | 0.3605 | 0.9318 | 0.9696 | 0.4783 | 1.9655 |
|  |  | IHGC | MR Egger | 9 | -1.1527 | 3.2020 | 0.7295 | 0.3158 | 0.0006 | 167.8709 |
|  |  |  | Weighted median | 9 | 0.0880 | 0.5857 | 0.8805 | 1.0920 | 0.3464 | 3.4420 |
|  |  |  | Inverse variance weighted | 9 | 0.1840 | 0.4909 | 0.7077 | 1.2021 | 0.4593 | 3.1462 |
| Computer use | MA | FinnGen | MR Egger | 70 | -1.6257 | 1.0056 | 0.1106 | 0.1968 | 0.0274 | 1.4125 |
|  |  |  | Weighted median | 70 | 0.1110 | 0.2461 | 0.6519 | 1.1174 | 0.6898 | 1.8102 |
|  |  |  | Inverse variance weighted | 70 | 0.1057 | 0.1833 | 0.5643 | 1.1115 | 0.7760 | 1.5920 |
|  |  | IHGC | MR Egger | 74 | 1.4423 | 1.3410 | 0.2857 | 4.2304 | 0.3054 | 58.5898 |
|  |  |  | Weighted median | 74 | 0.0602 | 0.3296 | 0.8550 | 1.0621 | 0.5567 | 2.0263 |
|  |  |  | Inverse variance weighted | 74 | 0.0571 | 0.2524 | 0.8211 | 1.0587 | 0.6455 | 1.7364 |
| Playing computer games | MA | FinnGen | MR Egger | 38 | -0.4773 | 1.4421 | 0.7426 | 0.6205 | 0.0367 | 10.4781 |
|  |  |  | Weighted median | 38 | -0.1390 | 0.3709 | 0.7080 | 0.8703 | 0.4206 | 1.8005 |
|  |  |  | Inverse variance weighted | 38 | -0.2827 | 0.2593 | 0.2755 | 0.7537 | 0.4534 | 1.2529 |
|  |  | IHGC | MR Egger | 47 | -0.4790 | 2.6777 | 0.8588 | 0.6194 | 0.0033 | 117.8392 |
|  |  |  | Weighted median | 47 | -0.5015 | 0.7282 | 0.4910 | 0.6056 | 0.1453 | 2.5239 |
|  |  |  | Inverse variance weighted | 47 | -0.4238 | 0.5319 | 0.4255 | 0.6545 | 0.2308 | 1.8564 |
| Watching television | MA | FinnGen | MR Egger | 81 | 1.0415 | 0.8621 | 0.2306 | 2.8334 | 0.5230 | 15.3505 |
|  |  |  | Weighted median | 81 | 0.1565 | 0.2441 | 0.5214 | 1.1694 | 0.7248 | 1.8869 |
|  |  |  | Inverse variance weighted | 81 | 0.0018 | 0.1681 | 0.9917 | 1.0018 | 0.7205 | 1.3927 |
|  |  | IHGC | MR Egger | 98 | 1.4305 | 0.9910 | 0.1522 | 4.1808 | 0.5993 | 29.1637 |
|  |  |  | Weighted median | 98 | 0.6708 | 0.3215 | 0.0369 | 1.9558 | 1.0415 | 3.6728 |
|  |  |  | Inverse variance weighted | 98 | 0.2968 | 0.2154 | 0.1681 | 1.3456 | 0.8823 | 2.0523 |
| UVMR, univariable mendelian randomization; SNPs, single-nucleotide polymorphisms; Beta, beta coefficient; Se, standard error; OR, odds ratio; lci, lower confidence interval; uci, upper confidence interval; MA, migraine with aura. | | | | | | | | | | |

**Supplementary Table 2.4. UVMR estimates for the associations between digital device use and migraine with no aura**

| Exposure | Outcome | Data source | Method | nSNPs | Beta | se | P-value | OR | OR_lci95 | OR_uci95 |
| --- | --- | --- | --- | --- | --- | --- | --- | --- | --- | --- |
| Mobile phone use | MO | FinnGen | MR Egger | 9 | -3.2556 | 1.8458 | 0.1211 | 0.0386 | 0.0010 | 1.4364 |
|  |  |  | Weighted median | 9 | 0.8174 | 0.4527 | 0.0710 | 2.2645 | 0.9324 | 5.4999 |
|  |  |  | Inverse variance weighted | 9 | 0.8091 | 0.3584 | **0.0240** | 2.2460 | 1.1126 | 4.5339 |
|  |  | IHGC | MR Egger | 9 | 0.3716 | 0.9394 | 0.7042 | 1.4500 | 0.2300 | 9.1414 |
|  |  |  | Weighted median | 9 | 0.2425 | 0.1988 | 0.2225 | 1.2744 | 0.8632 | 1.8815 |
|  |  |  | Inverse variance weighted | 9 | 0.2868 | 0.3547 | 0.0645 | 1.3321 | 0.6647 | 2.6697 |
| Computer use | MO | FinnGen | MR Egger | 65 | -0.7167 | 1.1327 | 0.5292 | 0.4883 | 0.0530 | 4.4964 |
|  |  |  | Weighted median | 65 | -0.4246 | 0.2833 | 0.1340 | 0.6541 | 0.3753 | 1.1397 |
|  |  |  | Inverse variance weighted | 65 | -0.4064 | 0.1928 | **0.0351** | 0.6661 | 0.4564 | 0.9720 |
|  |  | IHGC | MR Egger | 74 | -0.7893 | 1.1343 | 0.4888 | 0.4542 | 0.0492 | 4.1948 |
|  |  |  | Weighted median | 74 | 0.0358 | 0.2955 | 0.9036 | 1.0364 | 0.5808 | 1.8494 |
|  |  |  | Inverse variance weighted | 74 | -0.0285 | 0.2152 | 0.8948 | 0.9719 | 0.6375 | 1.4819 |
| Plays computer games | MO | FinnGen | MR Egger | 43 | 0.5816 | 1.8819 | 0.7588 | 1.7889 | 0.0447 | 71.5281 |
|  |  |  | Weighted median | 43 | -0.8392 | 0.5766 | 0.1456 | 0.4320 | 0.1395 | 1.3377 |
|  |  |  | Inverse variance weighted | 43 | -0.8972 | 0.4088 | **0.0282** | 0.4077 | 0.1830 | 0.9085 |
|  |  | IHGC | MR Egger | 47 | 0.8910 | 2.3385 | 0.7050 | 2.4377 | 0.0249 | 238.5277 |
|  |  |  | Weighted median | 47 | -0.2870 | 0.6285 | 0.6479 | 0.7505 | 0.2190 | 2.5723 |
|  |  |  | Inverse variance weighted | 47 | 0.1554 | 0.4645 | 0.7380 | 1.1681 | 0.4700 | 2.9033 |
| Watching television | MO | FinnGen | MR Egger | 85 | 1.0014 | 0.9214 | 0.2803 | 2.7220 | 0.4472 | 16.5666 |
|  |  |  | Weighted median | 85 | 0.5511 | 0.2840 | 0.0523 | 1.7352 | 0.9944 | 3.0278 |
|  |  |  | Inverse variance weighted | 85 | 0.7429 | 0.1831 | **4.98e-5** | 2.1020 | 1.4680 | 3.0097 |
|  |  | IHGC | MR Egger | 89 | 0.7179 | 0.9089 | 0.4318 | 2.7220 | 0.4472 | 16.5666 |
|  |  |  | Weighted median | 89 | 0.6285 | 0.2754 | **0.0225** | 1.7352 | 0.9944 | 3.0278 |
|  |  |  | Inverse variance weighted | 89 | 0.5447 | 0.2005 | **0.0066** | 2.1020 | 1.4680 | 3.0097 |
| UVMR, univariable mendelian randomization; SNPs, single-nucleotide polymorphisms; Beta, beta coefficient; Se, standard error; OR, odds ratio; lci, lower confidence interval; uci, upper confidence interval; MO, migraine with no aura. | | | | | | | | | | |

**Supplementary Table 2.5. Heterogeneity and Horizontal pleiotropy of MR analysis**

| Exposure | Data source | Outcome | Horizontal pleiotropy | | Heterogeneity test | | MR Steiger |
| --- | --- | --- | --- | --- | --- | --- | --- |
|  |  |  | MR PRESSO Global P-value | Egger_intercept P-value | IVW_Q_P-val | Egger_Q_P-val | Casual_direction test |
| Mobile phone use | Overall migraine | FinnGen | 0.6070 | 0.7142 | 0.5330 | 0.4239 | TRUE |
|  |  | IHGC | 0.3590 | 0.9211 | 0.3378 | 0.2498 | TRUE |
| Computer use | Overall migraine | FinnGen | 0.1050 | 0.7694 | 0.1164 | 0.1017 | TRUE |
|  |  | IHGC | 0.7050 | 0.3376 | 0.7203 | 0.7199 | TRUE |
| Playing computer games | Overall migraine | FinnGen | 0.8130 | 0.8917 | 0.8062 | 0.7717 | TRUE |
|  |  | IHGC | 0.7040 | 0.7562 | 0.5573 | 0.5143 | TRUE |
| Watching television | Overall migraine | FinnGen | 0.8750 | 0.6252 | 0.7083 | 0.6822 | TRUE |
|  |  | IHGC | 0.4580 | 0.9718 | 0.4445 | 0.4146 | TRUE |
| Mobile phone use | MA | FinnGen | 0.1090 | 0.1320 | 0.1109 | 0.2383 | TRUE |
|  |  | IHGC | 0.1560 | 0.6849 | 0.1426 | 0.1041 | TRUE |
| Computer use | MA | FinnGen | 0.1010 | 0.0846 | 0.1040 | 0.1427 | TRUE |
|  |  | IHGC | 0.0540 | 0.2964 | 0.0615 | 0.0639 | TRUE |
| Playing computer games | MA | FinnGen | 0.5790 | 0.8675 | 0.5280 | 0.5263 | TRUE |
|  |  | IHGC | 0.1520 | 0.9833 | 0.1625 | 0.1387 | TRUE |
| Watching television | MA | FinnGen | 0.7240 | 0.2225 | 0.7157 | 0.7316 | TRUE |
|  |  | IHGC | 0.4970 | 0.2441 | 0.4932 | 0.5040 | TRUE |
| Mobile phone use | MO | FinnGen | 0.2290 | 0.0607 | 0.2087 | 0.5525 | TRUE |
|  |  | IHGC | 0.1610 | 0.8878 | 0.6779 | 0.5747 | TRUE |
| Computer use | MO | FinnGen | 0.3150 | 0.7818 | 0.3018 | 0.2743 | TRUE |
|  |  | IHGC | 0.1610 | 0.4966 | 0.1706 | 0.1606 | TRUE |
| Playing computer games | MO | FinnGen | 0.3120 | 0.4253 | 0.3054 | 0.2933 | TRUE |
|  |  | IHGC | 0.2070 | 0.7496 | 0.2387 | 0.2112 | TRUE |
| Watching television | MO | FinnGen | 0.7754 | 0.7754 | 0.3546 | 0.3287 | TRUE |
|  |  | IHGC | 0.8455 | 0.8455 | 0.8711 | 0.8547 | TRUE |
| MR PRESSO, mendelian randomization pleiotropy residual sum and outlier; IVW, inverse variance weighted; MA, migraine with aura; MO, migraine with no aura. | | | | | | | |

**Supplementary Table 2.6. Heterogeneity and Horizontal pleiotropy of initial MR analysis**

| Exposure | Data source | Outcome | Horizontal pleiotropy | | Heterogeneity test | |
| --- | --- | --- | --- | --- | --- | --- |
|  |  |  | MR_PRESSO Global P-value | Egger_intercept P-value | IVW_Q_P-val | Egger_Q_P-val |
| Mobile phone use | Overall migraine | FinnGen | **0.0040** | 0.2363 | **0.0025** | **0.0078** |
|  |  | IHGC | 0.3590 | 0.9211 | 0.3378 | 0.2498 |
| Computer use | Overall migraine | FinnGen | **<0.0010** | 0.4055 | **2.87E-08** | **2.94E-08** |
|  |  | IHGC | **<0.0010** | 0.7192 | **4.77E-12** | **3.27E-12** |
| Playing computer games | Overall migraine | FinnGen | **<0.0010** | 0.7614 | **3.47E-05** | **2.47E-05** |
|  |  | IHGC | **<0.0010** | 0.8202 | **3.11E-06** | **2.08E-06** |
| Watching television | Overall migraine | FinnGen | **<0.0010** | 0.1454 | **1.93E-12** | **5.46E-12** |
|  |  | IHGC | **<0.0010** | 0.5967 | **3.66E-09** | **2.94E-09** |
| Mobile phone use | MA | FinnGen | 0.1090 | 0.1320 | 0.1109 | 0.2383 |
|  |  | IHGC | 0.1560 | 0.6849 | 0.1426 | 0.1041 |
| Computer use | MA | FinnGen | **0.0020** | 0.16932 | **0.0002** | **0.0003** |
|  |  | IHGC | 0.0540 | 0.2964 | 0.0615 | 0.0639 |
| Playing computer games | MA | FinnGen | **0.0460** | 0.3777 | **0.0479** | **0.0471** |
|  |  | IHGC | 0.1520 | 0.9833 | 0.1625 | 0.1387 |
| Watching television | MA | FinnGen | **<0.0010** | **0.0263** | **2.78E-07** | **1.91E-06** |
|  |  | IHGC | 0.4970 | 0.2441 | 0.4932 | 0.5040 |
| Mobile phone use | MO | FinnGen | 0.7240 | 0.2225 | 0.7157 | 0.7316 |
|  |  | IHGC | 0.1610 | 0.8878 | 0.6779 | 0.5747 |
| Computer use | MO | FinnGen | **0.001** | 0.6929 | **0.0013** | **0.0011** |
|  |  | IHGC | 0.1610 | 0.4966 | 0.1706 | 0.1606 |
| Playing computer games | MO | FinnGen | **0.016** | 0.5032 | **0.0118** | **0.0106** |
|  |  | IHGC | 0.2070 | 0.7496 | 0.2387 | 0.2112 |
| Watching television | MO | FinnGen | **<0.001** | 0.3502 | **0.0002** | **0.0002** |
|  |  | IHGC | **0.0200** | 0.5871 | **0.0154** | **0.0138** |
| MR PRESSO, mendelian randomization pleiotropy residual sum and outlier; IVW, inverse variance weighted; MA, migraine with aura; MO, migraine with no aura. | | | | | | |

**Supplementary Table 2.7. Estimates from MVMR between digital devices use and migraine**

| Exposure | Outcome | nSNPs | P-value | OR | OR_lci95 | OR_uci95 |
| --- | --- | --- | --- | --- | --- | --- |
| Stroke | Overall migraine | 3 | 0.4086 | 1.0519 | 0.9330 | 1.1859 |
| Moderate to vigorous physical activity levels | Overall migraine | 4 | 0.1616 | 1.3167 | 0.8958 | 1.935 |
| Hypertension | Overall migraine | 105 | 0.0693 | 1.4078 | 0.9734 | 2.0361 |
| Major depression | Overall migraine | 10 | **2.41E-05** | 1.4333 | 1.2128 | 1.6940 |
| Cigarettes smoked per day | Overall migraine | 4 | 0.8354 | 0.9898 | 0.8987 | 1.0902 |
| Alcoholic drinks per week | Overall migraine | 10 | 0.3389 | 0.8464 | 0.6013 | 1.1913 |
| Mobile phone use | Overall migraine | 2 | **0.0320** | 1.3966 | 1.0291 | 1.8954 |
| Body mass index (BMI) | Overall migraine | 245 | 0.0087 | 0.8469 | 0.7479 | 0.9589 |
| Insomnia | Overall migraine | 9 | 0.9887 | 1.0035 | 0.6191 | 1.6264 |
| Computer use | Overall migraine | 6 | 0.1537 | 1.4032 | 0.8810 | 2.2348 |
| Playing computer games | Overall migraine | 10 | 0.8083 | 1.0957 | 0.5236 | 2.2929 |
| Watching television | Overall migraine | 29 | **0.0011** | 2.0106 | 1.3180 | 3.0670 |
| MVMR, multivariable mendelian randomization study; SNPs, single-nucleotide polymorphisms; OR, odds ratio. | | | | | | |

**Supplementary Table 2.8. Estimates from MVMR between digital devices use and migraine with aura**

| Exposure | Outcome | nSNPs | P-value | OR | OR_lci95 | OR_uci95 |
| --- | --- | --- | --- | --- | --- | --- |
| Stroke | MA | 3 | 0.1236 | 1.1350 | 0.9660 | 1.3336 |
| Moderate to vigorous physical activity levels | MA | 4 | 0.0167 | 1.8814 | 1.1214 | 3.1564 |
| Hypertension | MA | 105 | 0.0842 | 1.5479 | 0.9427 | 2.5418 |
| Major depression | MA | 10 | 0.0195 | 1.3068 | 1.0440 | 1.6356 |
| Cigarettes smoked per day | MA | 4 | 0.8467 | 0.9873 | 0.8671 | 1.1241 |
| Alcoholic drinks per week | MA | 10 | 0.8752 | 1.0375 | 0.6553 | 1.6426 |
| Mobile phone use | MA | 2 | 0.1453 | 1.3565 | 0.8999 | 2.0448 |
| Body mass index (BMI) | MA | 245 | 0.0570 | 0.8503 | 0.7195 | 1.0048 |
| Insomnia | MA | 9 | 0.9078 | 1.0390 | 0.5434 | 1.9869 |
| Computer use | MA | 6 | 0.1549 | 1.5744 | 0.8424 | 2.9425 |
| Playing computer games | MA | 10 | 0.5430 | 0.7350 | 0.2724 | 1.9826 |
| Watching television | MA | 29 | 0.3059 | 1.3451 | 0.7626 | 2.3726 |
| MVMR, multivariable mendelian randomization study; SNPs, single-nucleotide polymorphisms; MA, migraine with aura. OR, odds ratio. | | | | | | |

**Supplementary Table 2.9. Estimates from MVMR between digital devices use and migraine with no aura**

| Exposure | Outcome | nSNPs | P-value | OR | OR_lci95 | OR_uci95 |
| --- | --- | --- | --- | --- | --- | --- |
| Stroke | MO | 3 | 0.9530 | 1.0054 | 0.8416 | 1.2010 |
| Moderate to vigorous physical activity levels | MO | 4 | 0.2984 | 1.3537 | 0.7649 | 2.3958 |
| Hypertension | MO | 105 | 0.5101 | 1.2018 | 0.6955 | 2.0768 |
| Major depression | MO | 10 | **1.24E-05** | 1.7374 | 1.3561 | 2.2258 |
| Cigarettes smoked per day | MO | 4 | 0.2887 | 1.0805 | 0.9365 | 1.2468 |
| Alcoholic drinks per week | MO | 10 | 0.0826 | 0.6385 | 0.3848 | 1.0596 |
| Mobile phone use | MO | 2 | **0.0062** | 1.8810 | 1.1963 | 2.9578 |
| Body mass index (BMI) | MO | 245 | 0.0005 | 0.7195 | 0.5986 | 0.8650 |
| Insomnia | MO | 9 | 0.8793 | 1.0572 | 0.5154 | 2.1684 |
| Computer use | MO | 6 | 0.3024 | 1.4375 | 0.7212 | 2.8651 |
| Playing computer games | MO | 10 | 0.4526 | 1.5212 | 0.5090 | 4.5461 |
| Watching television | MO | 29 | **6.99E-05** | 3.5627 | 1.9047 | 6.6640 |
| MVMR, multivariable mendelian randomization study; SNPs, single-nucleotide polymorphisms. MO, migraine with no aura. OR, odds ratio. | | | | | | |

**Supplementary Table 2.10. Removed outliers of UVMR in discovery corhort**

| Exposure | Outcome | Outliers |
| --- | --- | --- |
| Mobile phone use | Overall migraine | rs247584; rs4638225 |
| Computer use | Overall migraine | rs10208088;rs2761438;rs10518019;rs2041687;rs2068625;  rs2120461; rs2734833; rs3730399; rs707926; rs7335281 |
| Computer use | MA | rs10208088; rs2734833; rs28710456 |
| Computer use | MO | rs10208088;rs10828248;rs1229984;rs147543875;  rs1987942;rs6935828;rs707926; rs72828532 |
| Playing computer games | Overall migraine | rs62241000;rs3757589;rs4938017;rs12129719;rs12138787  rs144377835;rs35104374;rs6536378;rs7907706 |
| Playing computer games | MA | rs3757589;rs4938017 |
| Playing computer games | MO | rs11223780;rs11548535;rs12129719;rs35104374 |
| Watching TV | Overall migraine | rs11877758;rs34094119;rs7539775;rs75499503;rs10269099  rs11911112;rs1727332;rs180396;rs34811474;rs4788616;  rs494566;rs7089973;rs73571431;rs75641275;rs7708324  rs78227853;rs9471333 |
| Watching TV | MA | rs75499503;rs10109061;rs11714337;rs11877758;rs11911112  rs1727332;rs180396;rs2678662;rs34094119;rs35797019;  rs4110177;rs4303732;rs6814554;rs7539775;rs78227853 |
| Watching TV | MO | rs11714337;rs12214364;rs1727332;rs2725371; rs34811474;  rs6511708;rs7089973;rs73571431;rs7539775; rs78227853;  rs9300594 |
| UVMR, univariable Mendelian Randomization; MA, migraine with aura; MO, migraine with no aura. | | |

**Supplementary Table 2.11. Removed outliers of UVMR in validation corhort**

| Exposure | Outcome | Outliers |
| --- | --- | --- |
| Computer use | Overall migraine | rs113851275;rs72828532;rs9375188;rs113851275;rs72828532  rs9375188;rs1037091;rs10828248;rs112600282;rs11942953  rs12706626;rs13262595;rs1987942;rs2120461;rs3730399  rs707926;rs73578186;rs7526112;rs9537571 |
| Playing computer games | Overall migraine | rs35104374;rs12129719;rs11223780;rs13262595;rs159962  rs34402857;rs411844;rs55668363;rs56176431;rs62241000  rs9688977 |
| Watching TV | Overall migraine | rs11911112;rs7539775;rs11877758;rs1324491;rs2906604  rs4339469;rs4788616;rs62199883 ;rs6895658;rs75641275  rs7899206;rs9867437 |
| Watching TV | MO | rs1324491;rs17789218;rs2906604 ;rs4567133;rs4747438  rs7539775;rs78227853;rs996234 |
| UVMR, univariable Mendelian Randomization; MO, migraine with no aura. | | |
