# Supplementary material for: The role of digital device use on the risk of migraine: a univariable and multivariable Mendelian randomization study
Source: Front Neurol. 2024 Oct 30;15:1462414. doi: 10.3389/fneur.2024.1462414 (PMC11557339; doi:10.3389/fneur.2024.1462414)
Supplement: Supplementary file 4 [file Table_4.docx]

Supplementary Material

# Supplementary Data

Supplementary Material should be uploaded separately on submission. Please include any supplementary data, figures and/or tables.

Supplementary material is not typeset so please ensure that all information is clearly presented, the appropriate caption is included in the file and not in the manuscript, and that the style conforms to the rest of the article.

# Additional files

## Table 1_v2

Supplementary Table 1.1 STORBE-MR

## Table 2_v1

Supplementary Table 2.1 Details data sources of the GWAS included in the Mendelian randomization.

Supplementary Table 2.2. UVMR estimates for the associations between digital device use and overall migraine

Supplementary Table 2.3. UVMR estimates for the associations between digital device use and migraine with aura.

Supplementary Table 2.4. UVMR estimates for the associations between digital device use and migraine without aura.

Supplementary Table 2.5. Heterogeneity and Horizontal pleiotropy of MR analysis.

Supplementary Table 2.6. Heterogeneity and Horizontal pleiotropy of initial MR analysis.

Supplementary Table 2.7. Estimates from MVMR between digital device use and migraine.

Supplementary Table 2.8. Estimates from MVMR between digital device use and migraine with aura

Supplementary Table 2.9. Estimates from MVMR between digital device use and migraine with no aura.

Supplementary Table 2.10. Removed outliers of UVMR in discovery corhort.

Supplementary Table 2.11. Removed outliers of UVMR in validation corhort.

**
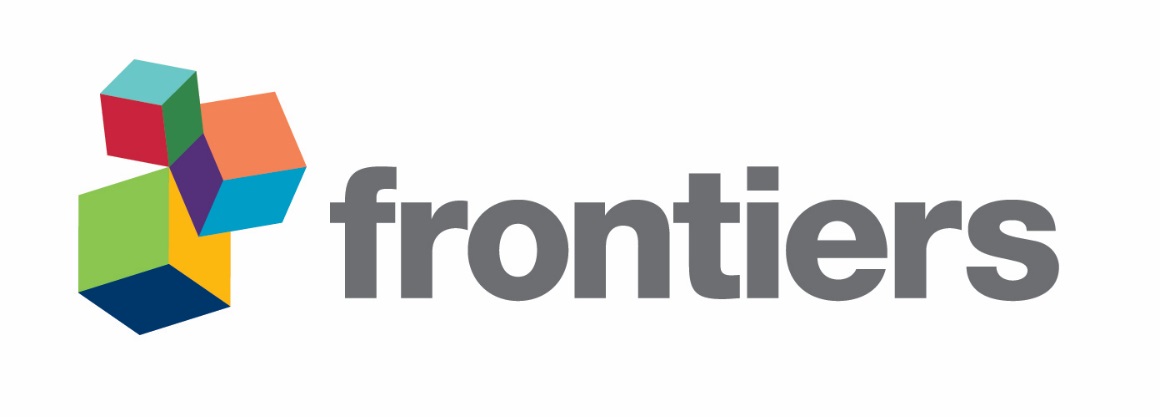
**

## Table 3_v1

Supplementary Table 3.1. Association of mobile phone use instrumental variants with risk of migraine across 2 databases.

Supplementary Table 3.2. Association of computer use instrumental variants with risk of migraine across 2 databases.

Supplementary Table 3.3. Association of playing computer games instrumental variants with risk of migraine across 2 databases.

Supplementary Table 3.4. Association of watching television instrumental variants with risk of migraine across 2 databases.

Supplementary Table 3.5. The members of the International Headache Genetics Consortium (IHGC).

## Image 1_v1

Supplementary Figure S1. MR Leave−One−Out analysis for assessing causal effect of digital devices use on overall migraine risk. This forest plot presents the results of the leave-one-out analysis, where each point estimate represents the causal effect of digital device use on migraine risk when one single nucleotide polymorphism (SNP) is removed from the analysis at a time. The x-axis shows the estimated causal effect, and the horizontal lines represent the 95% confidence intervals for each analysis. (A) Causal effects of mobile phone use on overall migraine from FinnGen; (B) Causal effects of computer use on overall migraine from FinnGen; (C) Causal effects of playing computer games on overall migraine from FinnGen; (D) Causal effects of computer use on overall migraine from FinnGen; (E) Causal effects of mobile phone use on overall migraine from IHGC; (F) Causal effects of computer use on overall migraine from IHGC; (G) Causal effects of playing computer games on overall migraine from IHGC; (H) Causal effects of computer use on overall migraine from IHGC.

Supplementary Figure S2. MR Leave−One−Out analysis for assessing causal effect of digital devices use on migraine with aura risk. This forest plot presents the results of the leave-one-out analysis, where each point estimate represents the causal effect of digital device use on migraine risk when one single nucleotide polymorphism (SNP) is removed from the analysis at a time. The x-axis shows the estimated causal effect, and the horizontal lines represent the 95% confidence intervals for each analysis. (A) Causal effects of mobile phone use on migraine with aura from FinnGen; (B) Causal effects of computer use on migraine with aura from FinnGen; (C) Causal effects of playing computer games on migraine with aura from FinnGen; (D) Causal effects of computer use on migraine with aura from FinnGen; (E) Causal effects of mobile phone use on migraine with aura from IHGC; (F) Causal effects of computer use on migraine with aura from IHGC; (G) Causal effects of playing computer games on migraine with aura from IHGC; (H) Causal effects of computer use on migraine with aura from IHGC.

Supplementary Figure S3. MR Leave−One−Out analysis for assessing causal effect of digital devices use on migraine with no aura risk. This forest plot presents the results of the leave-one-out analysis, where each point estimate represents the causal effect of digital devices use on migraine with no aura risk when one single nucleotide polymorphism (SNP) is removed from the analysis at a time. The x-axis shows the estimated causal effect, and the horizontal lines represent the 95% confidence intervals for each analysis. (A) Causal effects of mobile phone use on migraine with no aura from FinnGen; (B) Causal effects of computer use on migraine with no aura from FinnGen; (C) Causal effects of playing computer games on migraine with no aura from FinnGen; (D) Causal effects of computer use on migraine with no aura from FinnGen; (E) Causal effects of mobile phone use on migraine with no aura from IHGC; (F) Causal effects of computer use on migraine with no aura from IHGC; (G) Causal effects of playing computer games on migraine with no aura from IHGC; (H) Causal effects of computer use on migraine with no aura from IHGC.

Supplementary Figure S4. Scatter Plot of instrumental variable estimates for digital devices use and overall migraine risk. This scatter plot displays the instrumental variable estimates from the Mendelian randomization analysis. Each point represents a genetic variant used as an instrument, with the x-axis showing the effect of the variant on digital device use and the y-axis showing the effect of the variant on migraine risk. The line indicates the causal relationship estimated by the instrumental variables. (A) Causal effects of mobile phone use on overall migraine from FinnGen; (B) Causal effects of computer use on overall migraine from FinnGen; (C) Causal effects of playing computer games on overall migraine from FinnGen; (D) Causal effects of computer use on overall migraine from FinnGen; (E) Causal effects of mobile phone use on overall migraine from IHGC; (F) Causal effects of computer use on overall migraine from IHGC; (G) Causal effects of playing computer games on overall migraine from IHGC; (H) Causal effects of computer use on overall migraine from IHGC.

Supplementary Figure S5. Scatter Plot of instrumental variable estimates for digital devices use and migraine with aura risk. This scatter plot displays the instrumental variable estimates from the Mendelian randomization analysis. Each point represents a genetic variant used as an instrument, with the x-axis showing the effect of the variant on digital device use and the y-axis showing the effect of the variant on migraine risk. The line indicates the causal relationship estimated by the instrumental variables. (A) Causal effects of mobile phone use on migraine with aura from FinnGen; (B) Causal effects of computer use on migraine with aura from FinnGen; (C) Causal effects of playing computer games on migraine with aura from FinnGen; (D) Causal effects of computer use on migraine with aura from FinnGen; (E) Causal effects of mobile phone use on migraine with aura from IHGC; (F) Causal effects of computer use on migraine with aura from IHGC; (G) Causal effects of playing computer games on migraine with aura from IHGC; (H) Causal effects of computer use on migraine with aura from IHGC.

Supplementary Figure S6. Scatter Plot of instrumental variable estimates for digital devices use and migraine with no aura risk. This scatter plot displays the instrumental variable estimates from the Mendelian randomization analysis. Each point represents a genetic variant used as an instrument, with the x-axis showing the effect of the variant on digital device use and the y-axis showing the effect of the variant on migraine risk. The line indicates the causal relationship estimated by the instrumental variables. (A) Causal effects of mobile phone use on migraine with no aura from FinnGen; (B) Causal effects of computer use on migraine with no aura from FinnGen; (C) Causal effects of playing computer games on migraine with no aura from FinnGen; (D) Causal effects of computer use on migraine with no aura from FinnGen; (E) Causal effects of mobile phone use on migraine with no aura from IHGC; (F) Causal effects of computer use on migraine with no aura from IHGC; (G) Causal effects of playing computer games on migraine with no aura from IHGC; (H) Causal effects of computer use on migraine with no aura from IHGC.
