# Supplementary material for: The role of digital device use on the risk of migraine: a univariable and multivariable Mendelian randomization study
Source: Front Neurol. 2024 Oct 30;15:1462414. doi: 10.3389/fneur.2024.1462414 (PMC11557339; doi:10.3389/fneur.2024.1462414)
Supplement: Supplementary file 5 [file Image_1.pdf]

Supplementary Figure S1. MR Leave−One−Out analysis for assessing causal effect of digital devices use on overall migraine risk

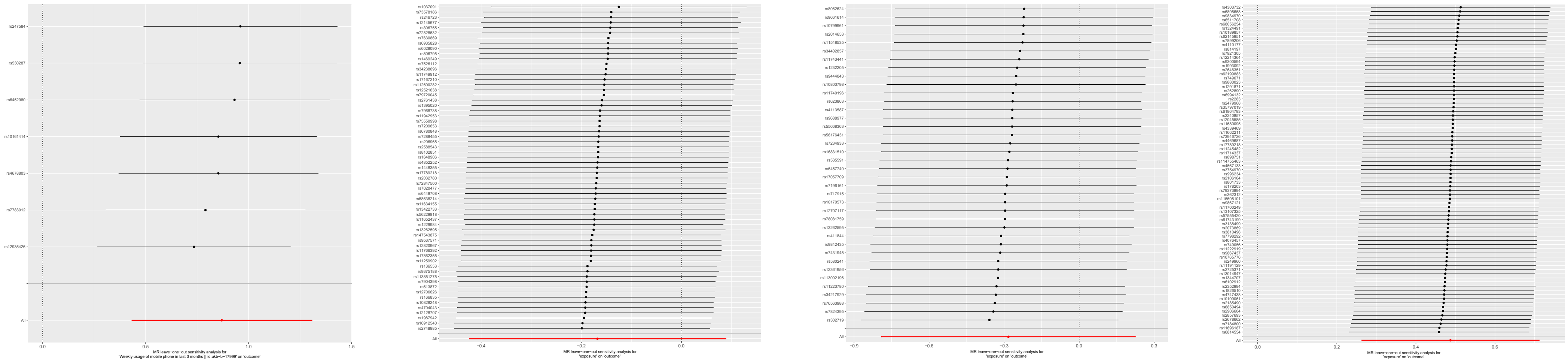

(A) Mobie phone use--FinnGen (B) Computer use--FinnGen (C) Playing computer games--FinnGen (D) Watching television--FinnGen

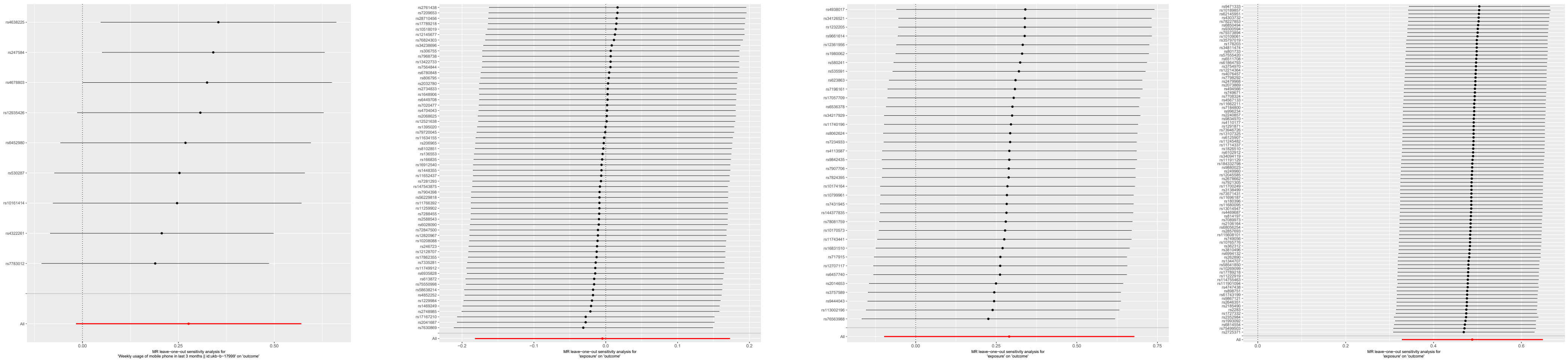

(E) Mobile phone use--IHGC (F) Computer use--IHGC (G) Playing computer games-IHGC (H) Watching television--IHGC

Supplementary Figure S2. MR Leave−One−Out analysis for assessing causal effect of digital devices use on migraine with aura risk

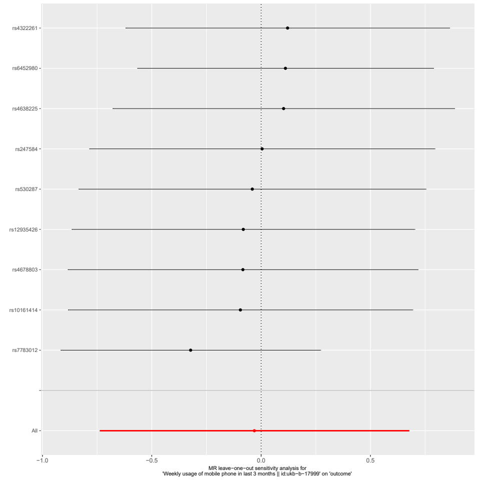

(A) Mobie phone use--FinnGen

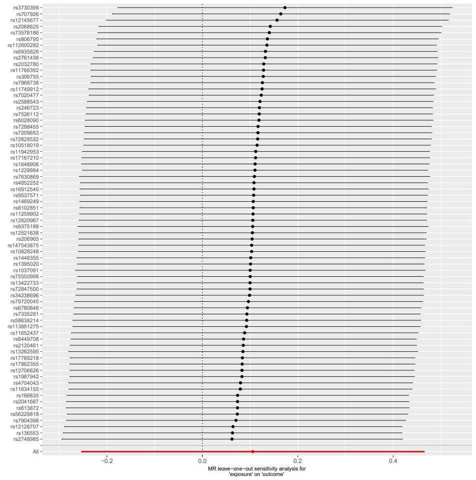

(B) Computer use--FinnGen

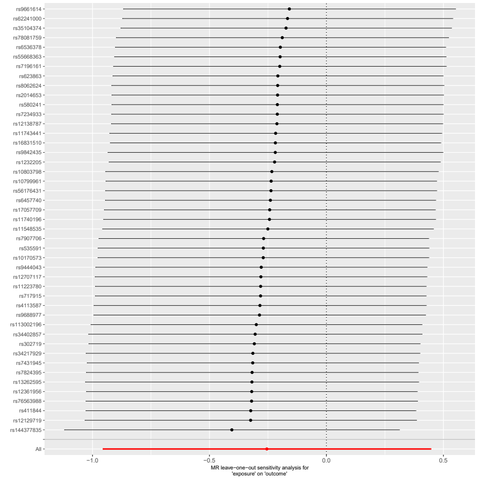

(C) Playing computer games--FinnGen

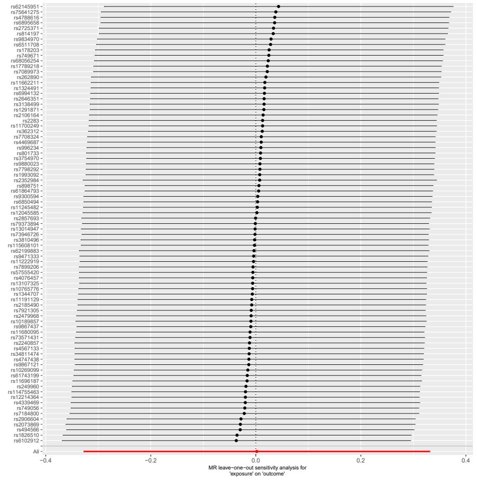

(D) Watching TV--FinnGen

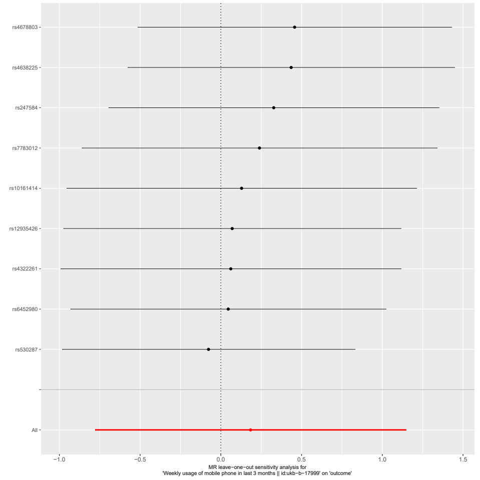

(E) Mobile phone use--IHGC

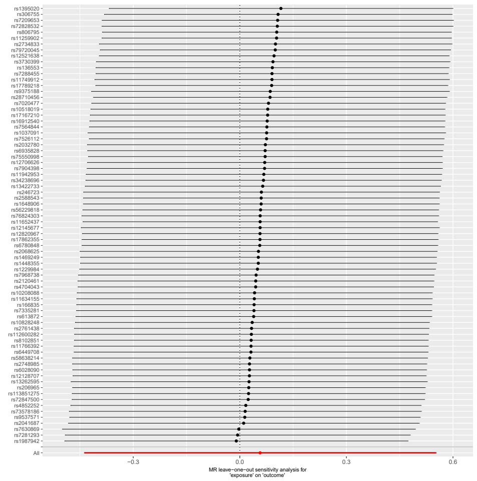

(F) Computer use--IHGC

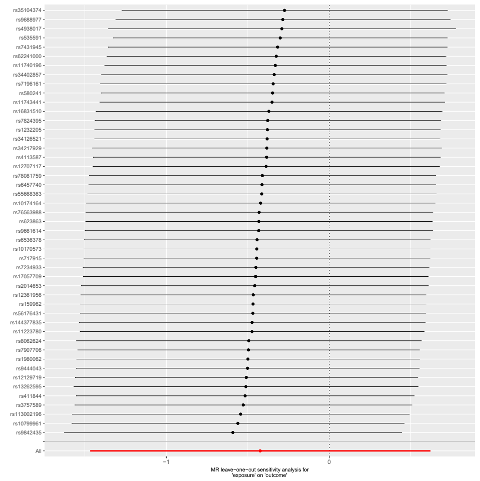

(G) Playing computer games-IHGC

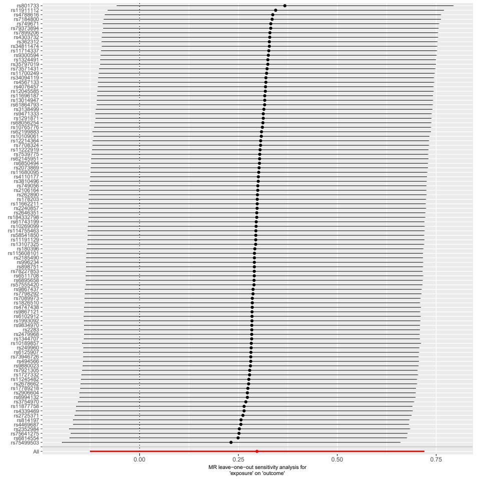

(H) Watching TV--IHGC

Supplementary Figure S3. MR Leave−One−Out analysis for assessing causal effect of digital devices use on migraine with no aura risk

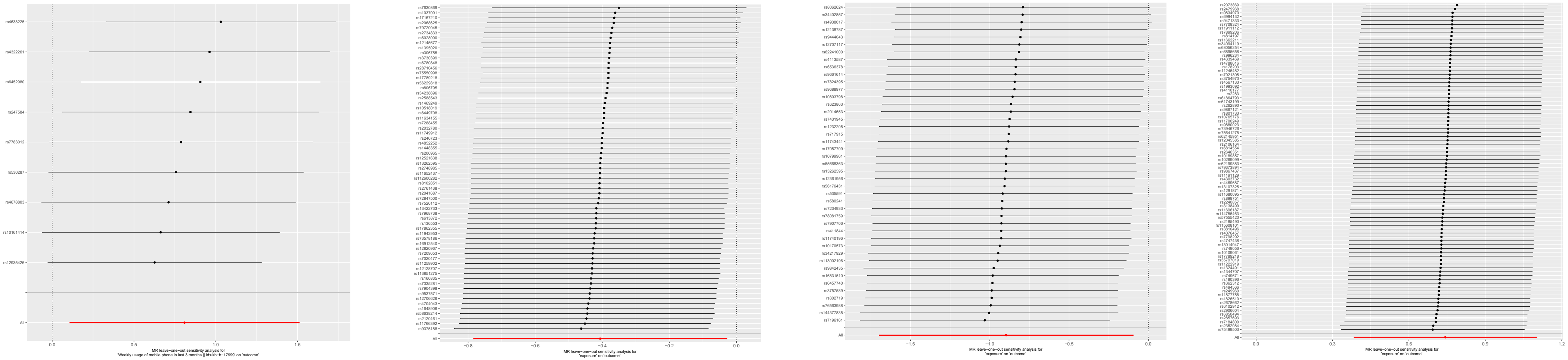

(A) Mobie phone use--FinnGen (B) Computer use--FinnGen (C) Plays computer games--FinnGen (D) Watching television--FinnGen

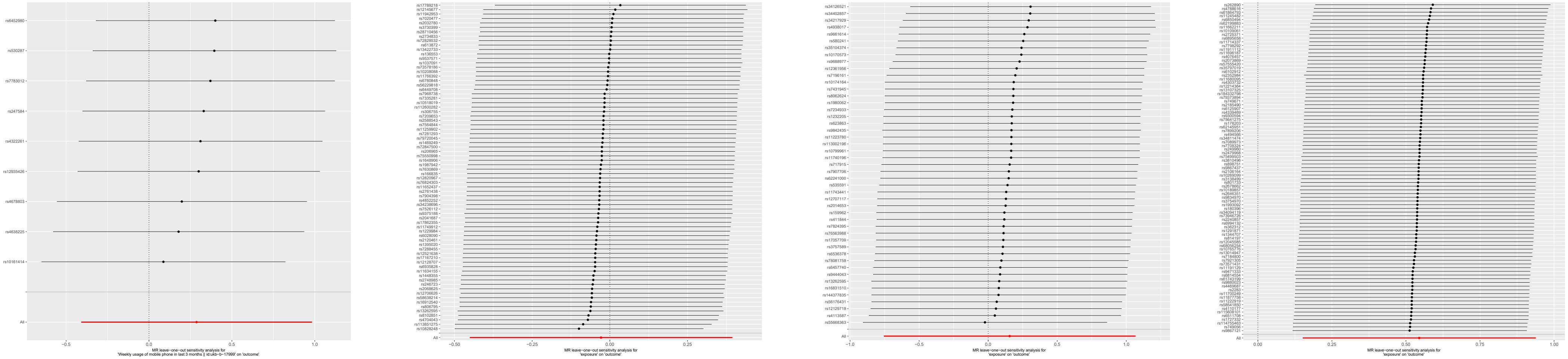

(E) Mobile phone use--IHGC (F) Computer use--IHGC (G) Playing computer games-IHGC (H) Watching television--IHGC

Supplementary Figure S4. Scatter Plot of instrumental variable estimates for digital devices use and overall migraine risk.

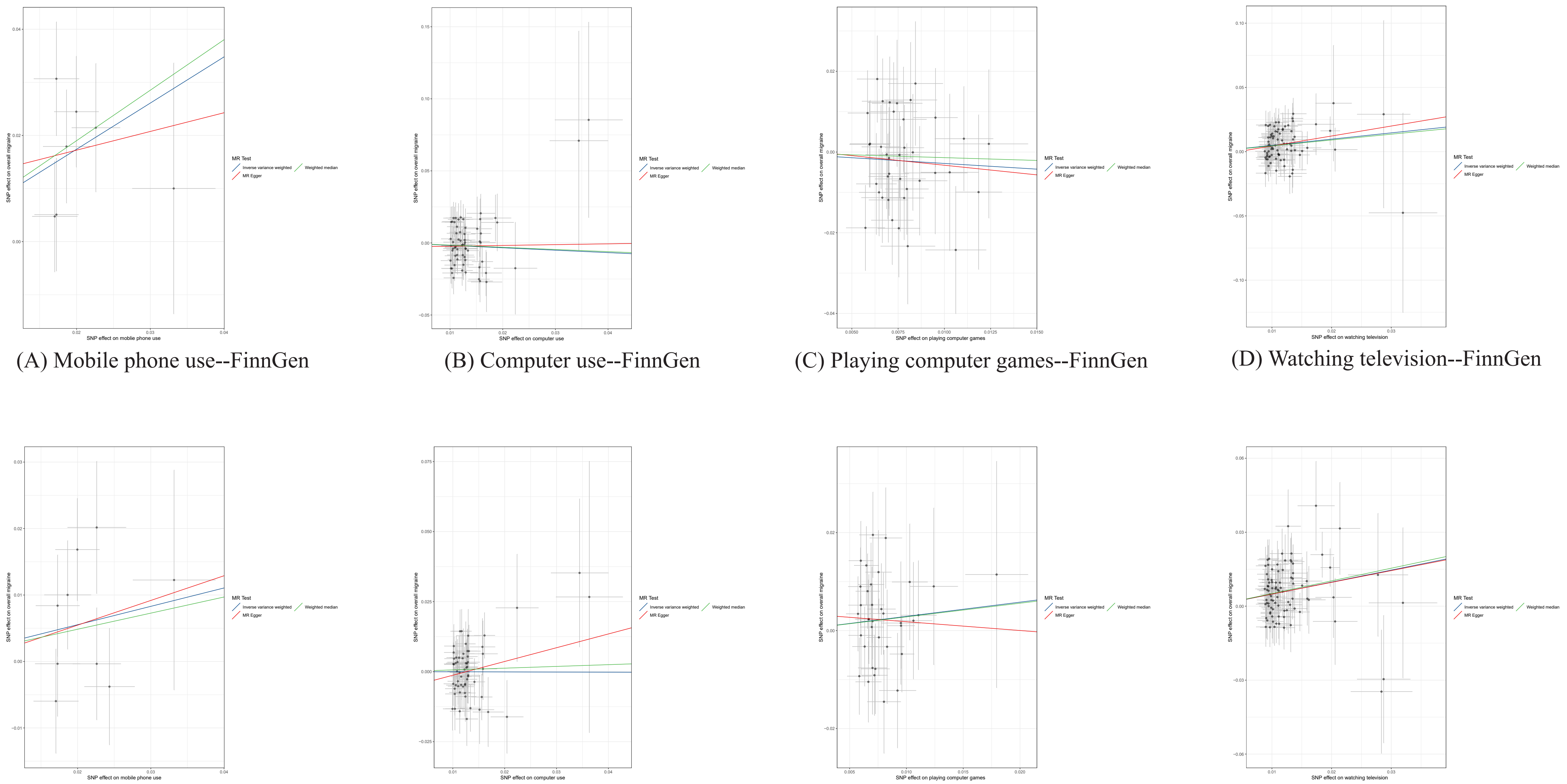

(A) Mobile phone use--FinnGen

(B) Computer use--FinnGen

(C) Playing computer games--FinnGen

(D) Watching television--FinnGen

(E) Mobile phone use--IHGC

(F) Computer use--IHGC

(G) Playing computer games--IHGC

(H) Watching television--IHGC

Supplementary Figure S5. Scatter Plot of instrumental variable estimates for digital devices use and migraine with aura risk

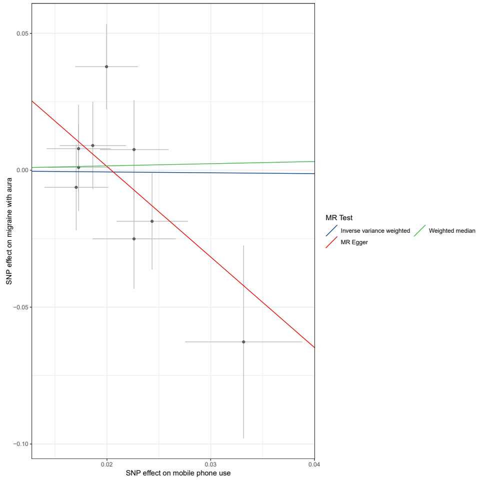

(A) Mobile phone use--FinnGen

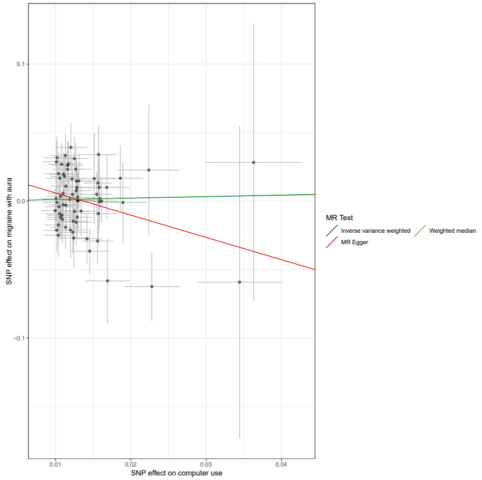

(B) Computer use--FinnGen

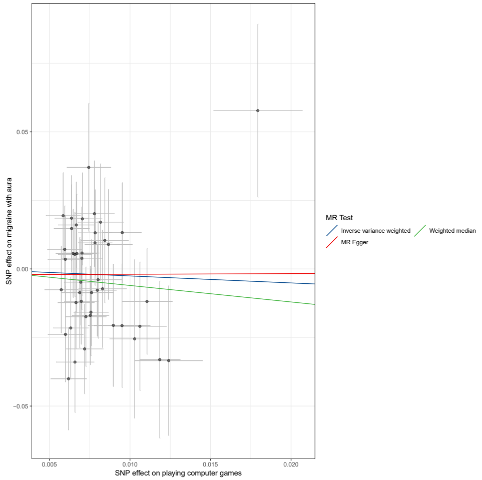

(C) Playing computer games--FinnGen

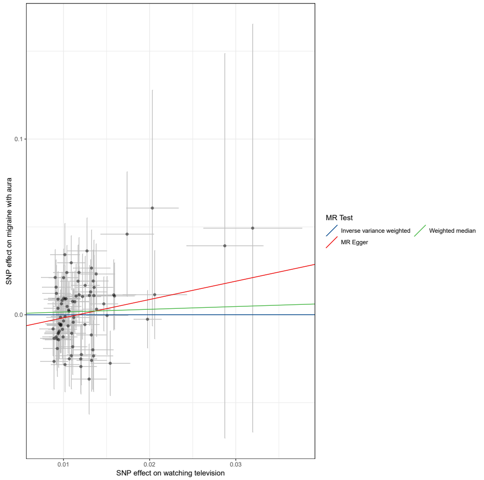

(D) Watching television--FinnGen

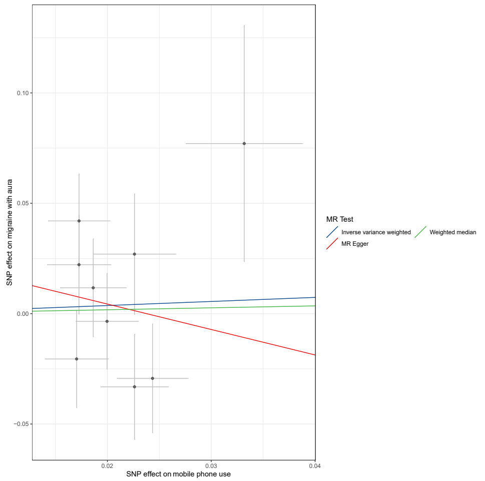

(E) Mobile phone use--IHGC

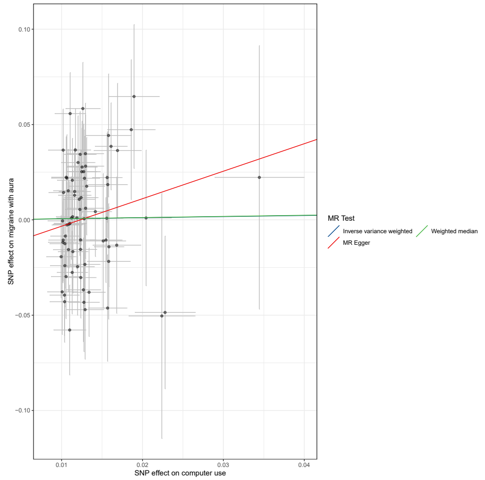

(F) Computer use--IHGC

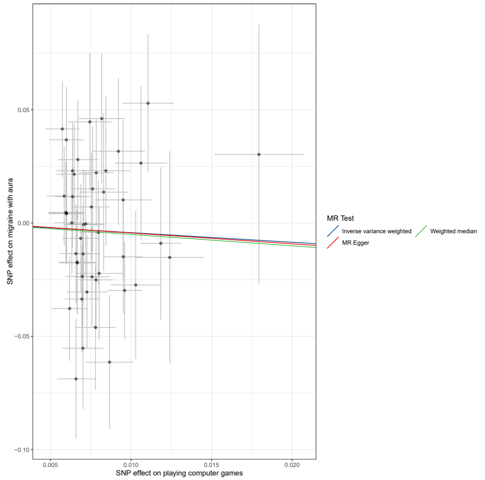

(G) Playing computer games--IHGC

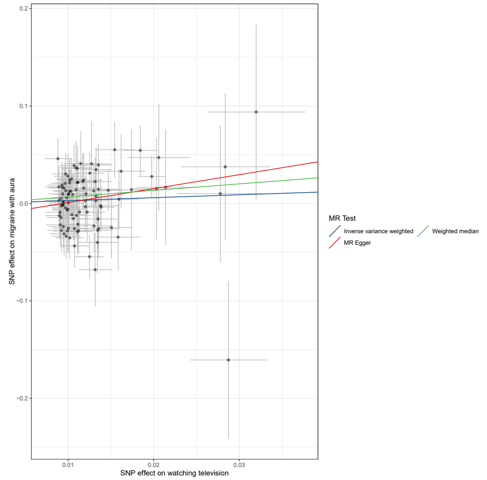

(H) Watching television--IHGC

Supplementary Figure S6. Scatter Plot of instrumental variable estimates for digital devices use and migraine with no aura risk

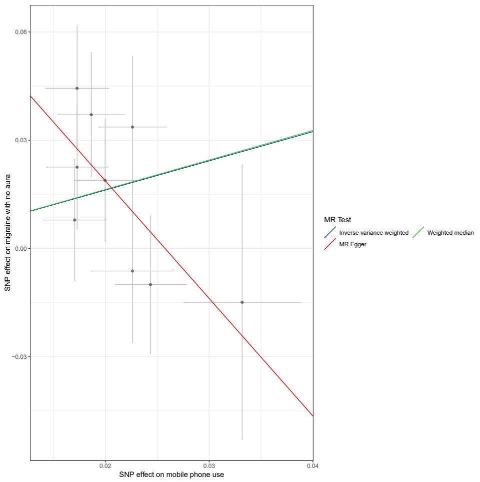

(A) Mobile phone use--FinnGen

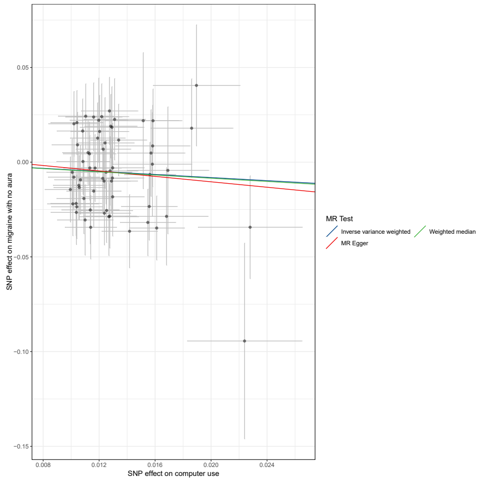

(B) Computer use--FinnGen

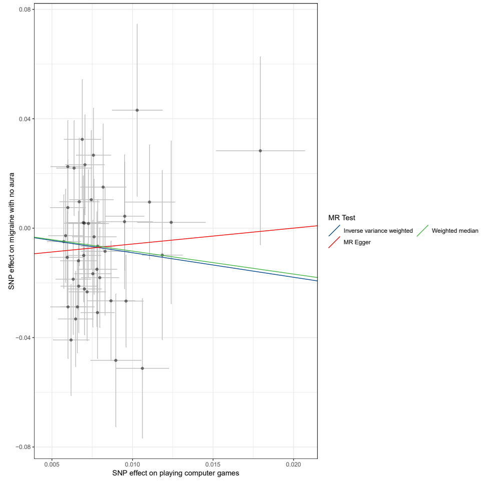

(C) Playing computer games--FinnGen

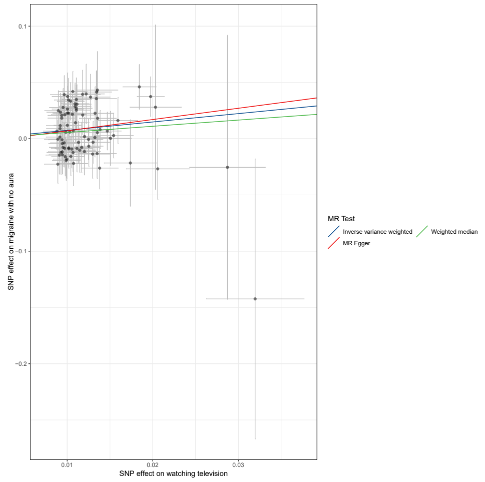

(D) Watching television--FinnGen

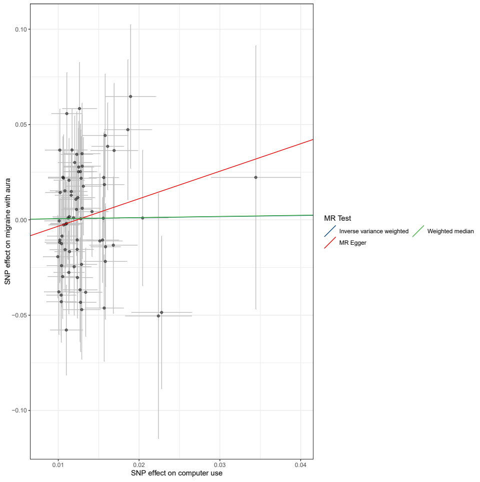

(E) Mobile phone use--IHGC

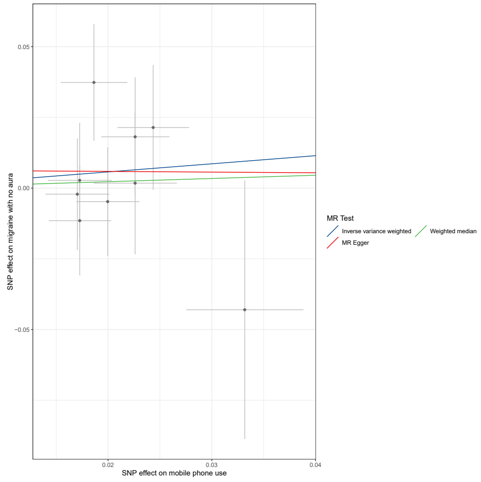

(F) Computer use--IHGC

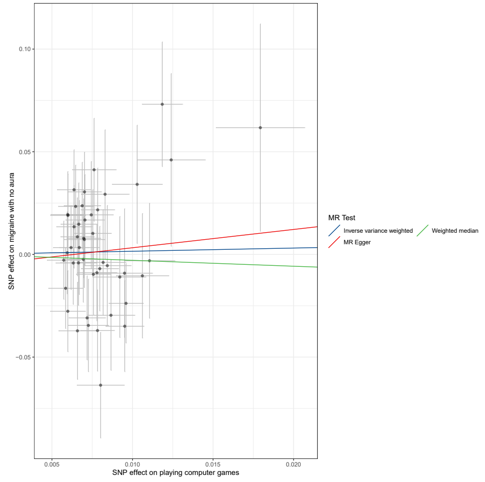

(G) Playing computer games--IHGC

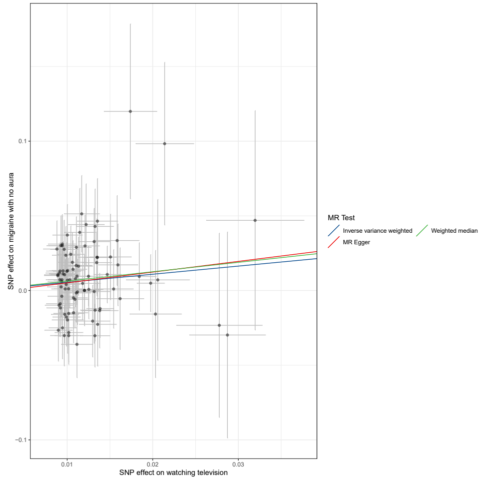

(H) Watching television--IHGC
